# Advanced age portends poorer prognosis after radical prostatectomy: a single center experience

**DOI:** 10.1007/s40520-022-02213-w

**Published:** 2022-08-17

**Authors:** Antonio Benito Porcaro, Alberto Bianchi, Sebastian Gallina, Emanuele Serafin, Giovanni Mazzucato, Stefano Vidiri, Damiano D’Aietti, Riccardo Rizzetto, Alessandro Tafuri, Clara Cerrato, Andrea Panunzio, Rossella Orlando, Davide Brusa, Matteo Brunelli, Salvatore Siracusano, Maria Angela Cerruto, Alessandro Antonelli

**Affiliations:** 1 Department of Urology, University of Verona, Azienda Ospedaliera Universitaria Integrata, Piazzale Stefani 1, 37126 Verona, Italy; 2 Department of Urology, Vito Fazzi Hospital, Lecce, Italy; 3 Department of Pathology, University of Verona, Azienda Ospedaliera Universitaria Integrata, Verona, Italy; 4 Department of Life, Health and Environmental Sciences, University of L’Aquila, L’Aquila, Italy

**Keywords:** Prostate cancer, Robot-assisted radical prostatectomy, Advanced age, Prostate cancer progression

## Abstract

**Introduction and objective:**

Although advanced age doesn’t seem to impair oncological outcomes after robot-assisted radical prostatectomy (RARP), elderly patients have increased rates of prostate cancer (PCa) related deaths due to a higher incidence of high-risk disease. The potential unfavorable impact of advanced age on oncological outcomes following RARP remains an unsettled issue. We aimed to evaluate the oncological outcome of PCa patients > 69 years old in a single tertiary center.

**Materials and methods:**

1143 patients with clinically localized PCa underwent RARP from January 2013 to October 2020. Analysis was performed on 901 patients with available follow-up. Patients ≥ 70 years old were considered elderly. Unfavorable pathology included ISUP grade group > 2, seminal vesicle, and pelvic lymph node invasion. Disease progression was defined as biochemical and/or local recurrence and/or distant metastases.

**Results:**

243 cases (27%) were classified as elderly patients (median age 72 years). Median (IQR) follow-up was 40.4 (38.7–42.2) months. Disease progression occurred in 159 cases (17.6%). Elderly patients were more likely to belong to EAU high-risk class, have unfavorable pathology, and experience disease progression after surgery (HR = 5.300; 95% CI 1.844–15.237; *p* = 0.002) compared to the younger patients.

**Conclusions:**

Elderly patients eligible for RARP are more likely to belong to the EAU high-risk category and to have unfavorable pathology that are independent predictors of disease progression. Advanced age adversely impacts on oncological outcomes when evaluated inside these unfavorable categories. Accordingly, elderly patients belonging to the EAU high-risk should be counseled about the increased risk of disease progression after surgery.

**Supplementary Information:**

The online version contains supplementary material available at 10.1007/s40520-022-02213-w.

## Introduction

Prostate cancer (PCa) is the second most diagnosed cancer in men worldwide with incidence mainly dependent on age and influenced by the usage of prostate-specific antigen (PSA) testing [1]. Robot-assisted radical prostatectomy (RARP) with or without extended pelvic lymph node dissection (ePLND) is the most performed procedure in clinically localized disease and it is increasingly applied also to elderly patients who are aged ≥ 70 years [1, 2]. Treating clinically localized PCa with RARP is feasible and safe in elderly patients; however, functional outcomes related to urinary continence and erectile function are worse when compared to younger cases [3–7]. Although advanced age does not seem to impair oncological outcomes after RARP, elderly patients have increased rates of PCa-related deaths for a higher incidence of advanced disease [1–6, 8–10]. The potential unfavorable impact of advanced age on oncological outcomes following RARP remains an unsettled issue. This study aims to evaluate the oncological outcome of elderly PCa patients aged ≥ 70 years in a single tertiary referral center.

## Materials and methods

### Selection of patients, data collection, and evaluation

Institutional Review Board approval was obtained from Azienda Ospedaliera Universitaria Integrata of Verona's ethical committee. Informed consent was obtained by all subjects. Data were collected prospectively but evaluated retrospectively. In a period ranging from January 2013 to October 2020, 1143 patients with clinically localized PCa were treated with RARP. Analysis was performed on 901 patients with available follow-up. Prostate-specific antigen (PSA; ng/mL), age (years), body mass index (BMI; kg/m^2^), prostate volume (PV; mL), percentage of biopsy positive cores (BPC), and the percentage ratio of positive and total taken cores (%) were evaluated for each case. PV was calculated by transrectal ultrasound (TRUS) standard methods. Biopsies performed elsewhere were assessed for the number of cores taken, tumor grade, and PV, which was measured by trans-rectal approach. In our Institution, the 14-core trans perineal technique was used. Patients were classified into risk classes, as recommended by EAU guidelines [1]. Preoperative physical status was evaluated by the American Society of Anesthesiologists (ASA) system [11]. RARP surgery was performed by experienced surgeons. ePLND was performed according to guidelines [1, 2]. Dissected lymph nodes were submitted in separate packages according to a standard anatomical template including external iliac, internal iliac plus obturator, Marcille’s common iliac, and Cloquet’s nodal stations, bilaterally [12]. Prostates were weighted and tumors were graded by the dedicated pathologist according to the International Society of Urologic Pathology (ISUP) system [1, 2]. Tumor quantitation was assessed as tumor load (TL), which was defined as the percentage of prostate involved by cancer; specifically, our dedicated pathologist assessed tumor quantitation by visual estimation of all the glass slides after all microscopically identifiable foci of carcinoma have been circled with a marked pen, as considered by ISUP association [13]. Surgical margins were stated positive when cancer invaded the inked surface of the specimen. Removed lymph nodes were counted and assessed for cancer invasion. Prostate surgical specimens were staged by the latest TNM system (8th edition)[1, 2]. Perioperative outcomes were evaluated for operating time, estimated intraoperative blood loss, nerve-sparing surgery, high and low volume surgeons, length of hospital stay (LOHS), and hospital readmission after discharge. Postoperative complications were coded according to the Clavien–Dindo system [12].

### Oncological and survival outcomes

Elderly patients were classified as ≥ 70 years of age. Unfavorable pathology included ISUP grade group > 2, seminal vesicle invasion (SVI), and pelvic lymph node invasion (PLNI). Patients were followed-up as recommended [1]. At PSA persistence/recurrence, imaging modalities were considered to restage the disease. The primary endpoint was disease progression, which was defined as biochemical recurrence and/or local recurrence and/or distant metastases. Biochemical recurrence after surgery was defined as a second confirmatory level of PSA ≥ 0.2 ng/mL [1].

### Statistical methods

Continuous variables were measured for medians and interquartile ranges (IQR). Categorical factors were assessed for frequencies (percentages). Associations of clinical, pathological, and perioperative factors with age (dichotomized into < vs ≥ 70 years) were assessed by logistic regression models (univariable and multivariable analysis).

The length of time between surgery and the clinical outcome of interest (PCa progression) or the last follow-up was measured as time to event occurrence. Univariable and multivariable Cox proportional hazards models estimated the association of factors (EAU risk class, BCP ≥ 50%, ISUP > 2, SVI, PLNI, positive surgical margins) with the risk of PCa progression; hazard ratios and relative 95% confidence intervals (CI) were evaluated. Satisfaction of proportional hazard model assumptions were evaluated graphically, plotting residuals to verify the linear relationship between the log hazard and each covariate. Models were also stratified by age groups to determine the effect modification of age on disease progression. Appropriate survival risk curves were generated. The software used to run the analysis was IBM-SPSS version 26. All tests were two-sided with *p* < 0.05 considered to indicate statistical significance.

## Results

### Patient demographics and perioperative outcomes

During the study period, a total of 1143 cases with clinically localized PCa treated with RARP were included in the initial study phase. Analysis was performed on 901 patients with available follow-up. The preliminary analysis of the 242 patients excluded found no statistically significant differences in terms of demographic characteristics compared to included patients.

Demographics of the population and subgroups of patients included for final analysis are reported in Table 1. Overall, 243 subjects (27%) were elderly men with a median age of 72 years. Elderly patients were more likely to have an impaired physical status (ASA > 2), to bear larger prostates, and to associate with higher-grade cancers (ISUP > 3). In the surgical specimen, elderly patients were more likely to have larger prostates, higher tumor grades (ISUP > 2), and tumor stages with SVI, as shown in Supplementary Table S1. Elderly patients were more likely to have higher rates of grade 1 Clavien–Dindo postoperative complications, but no longer LOHS or higher readmission rates after discharge (see supplementary Table S2). The distribution of EAU risk classes stratified by age groups is depicted in Supplementary Fig. 1, which shows that elderly patients were more likely to belong to the high-risk class because of the association with high-grade cancers.

**Table 1:** Demographics of the patient population stratified by age groups

Number	Age < 70 years	Age > / = 70 years	Univariable analysis		Multivariable analysis	
	658	243	OR (95% CI)	*P-*value	OR (95% CI)	*P-*value
Age (years)	63 (58–66)	72 (71–74)				
Body mass index; BMI (kg/m^2^)	25.7 (23.8–28.1)	25.7 (24–28.1)	0.991 (0.947–1.037)	0.693	0.973 (0.927–1.020)	0.255
*ASA system*						
ASA 1	64 (9.7)	13 (5.3)	Ref		Ref	
ASA 2	542 (82.4)	201 (82.7)	1.621 (0.905–2.907)	0.105	1.583 (0.875–2.863)	0.129
ASA 3	52 (7.9)	29 (12)	2.454 (1.193–5.049)	**0.015**	2.397 (1.138–5.049)	**0.022**
Prostate volume; PV (mL)	39.5 (30–49.5)	40 (30–55)	1.011 (1.003–1.019)	**0.009**	1.012 (1.003–1.020)	**0.006**
*Prostate specific antigen; PSA (ng/mL)*						
PSA < 10 ng/mL	534 (81.2)	197 (81.1)	Ref		Ref	
PSA: 10–20 ng/mL	96 (14.6)	36 (14.8)	1.016 (0.670–1.542)	0.939	0.864 (0.557–1.339)	0.513
PSA > 20 ng/mL	28 (4.3)	10 (4.1)	0.968 (0.462–2.030)	0.932	0.662 (0.301–1.453)	0.303
*Percentage of biopsy positive cores; BPC (%)*						
BPC < 50%	494 (75.1)	174 (71.6)	Ref		Ref	
BPC ≥ 50%	164 (24.9)	69 (28.4)	1.194 (0.859–1.291)	0.346	1.180 (0.830–1.677)	0.357
*ISUP grade group*						
ISUP 1	263 (40)	80 (32.9)	Ref		Ref	
ISUP 2/3	330 (50.2)	116 (47.7)	1.156 (0.833–1.604)	0.387	1.168 (0.834–1.637)	0.367
ISUP 4/5	65 (9.9)	47 (19.3)	2.377 (1.514–3.732)	** < 0.0001**	2.585 (1.566–4.269)	** < 0.0001**
*Clinical stage (cT)*						
cT1	395 (60)	147 (60.5)	Ref		Ref	
cT2	244 (37.1)	89 (36.6)	0.980 (0.720–1.333)	0.898	0.842 (0.608–1.167)	0.301
cT3	17 (2.9)	7 (2.9)	0.990 (0.408–2.404)	0.982	0.712 (0.275–1.846)	0.485
*Clinical nodal stage (cN)*						
cN0	622 (94.5)	229 (94.2)	Ref		Ref	
cN1	36 (5.5)	14 (5.8)	1.056 (0.559–1.995)	0.866	0.891 (0.450–1.764)	0.741

Continuous variables are reported as medians (IQR, interquartile ranges) and categorical factors as frequency (percentage)

*ISUP* International Society of Urologic Pathology tumor grade group formulation, *OR* Odds ratio, *CI* confidence interval

### Effect modification of age on PCa progression

**Fig. 1:**
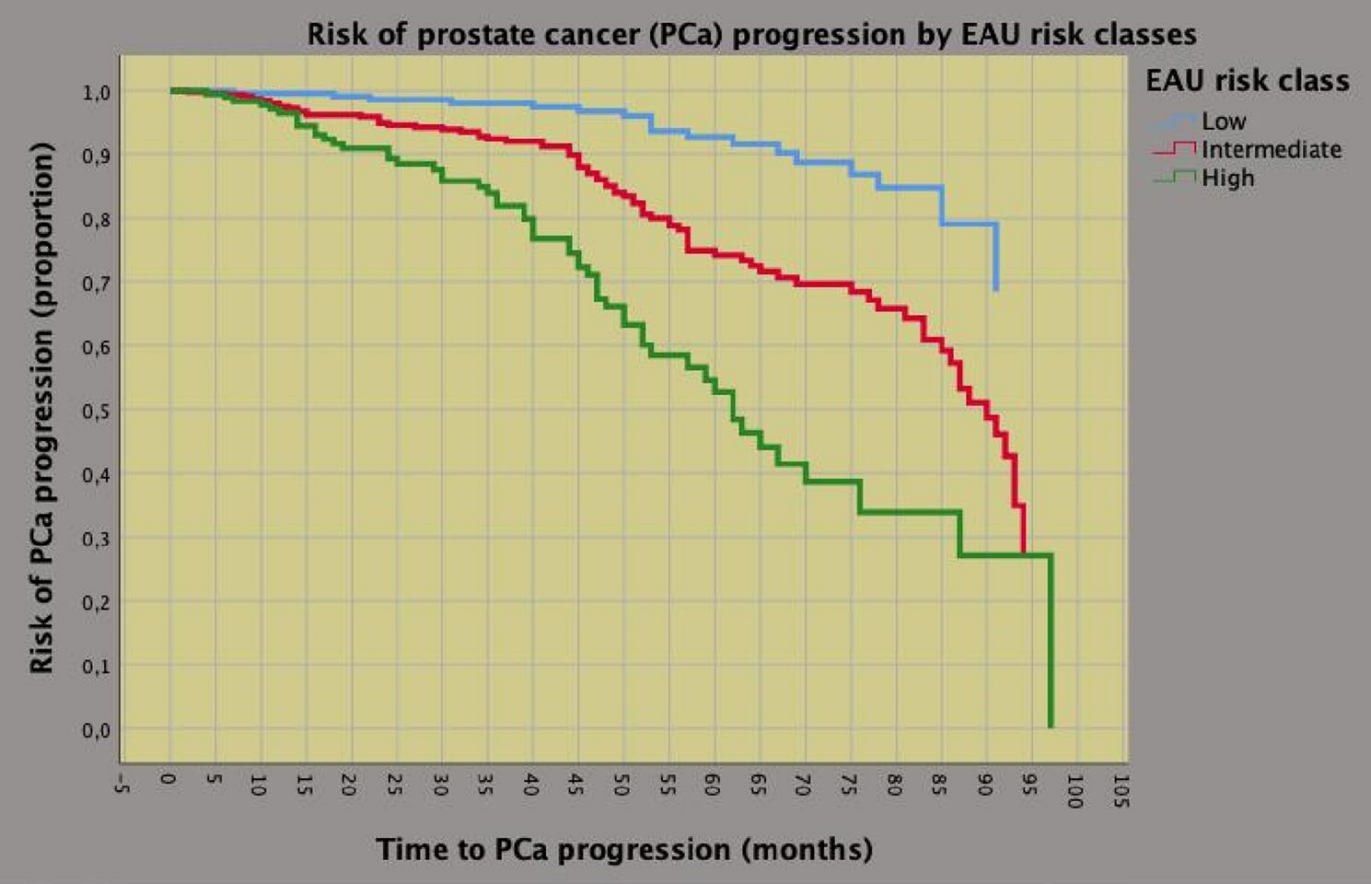
Risk curves of time to prostate cancer (PCa) progression stratified by clinical risk classes according to the European Society of Urology (EAU). As shown, the median time to disease progression was 62 months for the high-risk class and 90 months for the intermediate-risk class, but not reached for the low-risk class. See Table 3 for details

**Fig. 2:**
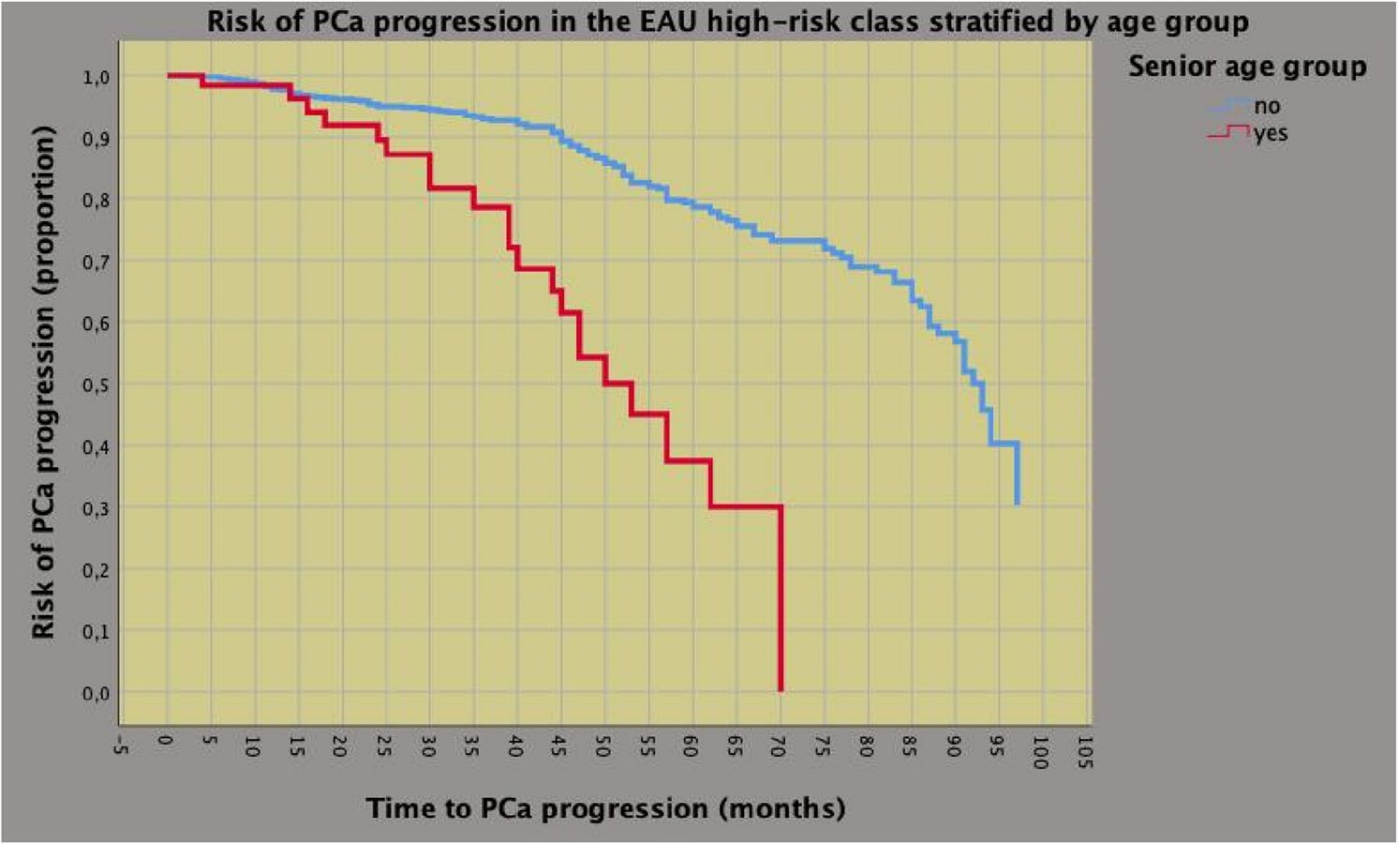
The adverse interacting effect of senior age on prostate cancer (PCa) progression in high-risk patients treated with robot-assisted radical prostatectomy (RARP). As illustrated, elderly patients were more likely to progress compared with younger subjects. Median time to disease progression was only 50 months for the senior age group and 92 months for younger cases. On univariable analysis, there was an adverse interaction effect of advanced age (hazard ratio, HR = 3.974; 95% CI 2.480–6.367; *p* < 0.0001) on disease progression, which was confirmed on multivariable analysis after adjusting for clinical factors. See also Table 3

Median (IQR) follow-up was 40.4 (38.7–42.2) months. Deaths occurred in 12 patients (overall survival 98.7%) of whom 4 related to PCa (cancer-specific survival 99.6%). Disease progression occurred in 159 cases (17.6%). Associations of clinical and pathological factors with the risk of PCa progression are reported in Table 2 and Supplementary Table S3. Patients presenting with unfavorable tumor features were more likely to progress after surgery. Subjects with unfavorable pathology were also more likely to progress after RARP. Table 3 shows univariable and multivariable risk models predicting PCa progression of population and age groups. As expected, EAU risk groups and unfavorable disease are associated with the risk of PCa progression. However, elderly patients belonging to the high-risk class were more likely to experience disease progression (HR = 5.029; 95% CI 1.685–15.010; *p* = 0.004) than younger patients (HR = 3.487; 95% CI 1.776–6.487; *p* < 0.0001); moreover, older subjects with unfavorable tumor grade and LNI were also more likely to experience PCa progression when compared with younger cases. Although the worst prognosis was predicted by the EAU high-risk class (Fig. 1), elderly patients were still more likely to progress compared to younger patients (Fig. 2 and Supplementary Fig. 2).

**Table 2:** Clinical factors predicting prostate cancer (PCa) progression in 901 patients treated with robot-assisted radical prostatectomy

Number of cases (%)	No PCa progression	PCa progression	Univariable analysis		Multivariable analysis	
	742 (82.4)	159 (17.6)	HR (95% CI)	*P*-value	HR (95% CI)	*P-*value
Age < 70 years	544 (73.3)	114 (71.7)	Ref		Ref	
Age ≥ 70 years	198 (26.7)	45 (28.3)	1.276 (0.901–1.806)	0.178	1.230 (0.861–1.757)	0.256
Body mass index; BMI (kg/m^2)	25.8 (23.9–28.1)	25.6 (24–28)	0.991 (0.942–1.043)	0.730	0.976 (0,929–1,026)	0.336
ASA 1	59 (8.0)	18 (11.3)	Ref		Ref	
ASA 2	615 (82.9)	128 (80.5)	0.671 (0.418–1.077)	0.098	0.920 (0.566–1.495)	0.737
ASA 3	68 (9.2)	13 (8.2)	0.762 (0.379–1.532)	0.445	0.782 (0.384–1.591)	0.497
PV	40 (30–50)	39 (30–50)	1.003 (0.994–1.012)	0.550	1.003 (0.993–1.013)	0.583
PSA < 10 ng/mL	631 (85)	100 (62.9)	Ref		Ref	
PSA: 10–20 ng/mL	92 (12.4)	40 (25.2)	2.524 (1.746–3.650)	** < 0.0001**	1.935 (1.301–2.879)	** < 0.0001**
PSA > 20 ng/mL	19 (2.6)	19 (11.9)	4.291 (2.620–7.030)	** < 0.0001**	2.420 (1.397–4.192)	**0.002**
BPC < 50%	572 (77.1)	96 (60.4)	Ref		Ref	
BPC ≥ 50%	170 (22.9)	63 (39.6)	2.225 (1.616–3.062)	** < 0.0001**	1.654 (1.164–2.353)	**0.005**
ISUP 1	300 (40.4)	43 (27)	Ref		Ref	
ISUP 2/3	366 (49.3)	80 (50.3)	2.653 (1.823–3.863)	** < 0.0001**	2.473 (1.685–3.630)	** < 0.0001**
ISUP 4/5	76 (10.2)	36 (22.6)	4.447 (2.843–6.954)	** < 0.0001**	2.480 (1.516–4.056)	** < 0.0001**
cT1	456 (61.5)	86 (54.1)	Ref		Ref	
cT2/3	286 (38.5)	73 (45.9)	1.902 (1.389–2.605)	** < 0.0001**	1.522 (1.098–2.110)	**0.012**
cN0	708 (95.4)	143 (89.9)	Ref		Ref	
cN1	34 (4.6)	16 (10.1)	3.300 (1.959–5.559)	** < 0.0001**	2.183 (1.257–3.794)	**0.006**

see Table 1; *HR* Hazard ratio, *CI* confidence interval

**Table 3:** Univariable and multivariable risk models of prostate cancer progression of population and age subgroups including patients treated with robot-assisted radical prostatectomy

Clinical model	Population (*n* = 901)		Age < 70 years (*n* = 658)		Age > / = 70 years (*n* = 243)	
	HR (95% CI)	*P*-value	HR (95% CI)	*P*-value	HR (95% CI)	*P*-value
*EAU Intermediate-risk class*						
Univariable analysis	2.307 (1.379–3.862)	**0.001**	2.434 (1.345–4.404)	**0.003**	1.955 (0.692–5.528)	0.206
Multivariable analysis	2.143 (1.276–3.600)	**0.004**	2.200 (1.209–4.006)	**0.010**	1.931 (0.681–5.472)	0.216
*EAU high-risk class*						
Univariable analysis	4.590 (2.632–8.004)	**< 0.0001**	4.224 (2.182–8.176)	**< 0.0001**	5.300 (1.814–15.237)	**0.002**
Multivariable analysis	3.848 (2.177–6.802)	**< 0.0001**	3.487 (1.776–6.487)	**< 0,0001**	5.029 (1.685–15.010)	**0.004**
*BPC ≥ 50%*						
Univariable analysis	2.208 (1.539–3.168)	**< 0.0001**	2.442 (1.595–3.740)	**< 0.0001**	1.705 (0.863–3.367)	0.125
Multivariable analysis	1.780 (1.244–2.590)	**0.003**	2.062 (1.352–3.194)	**0.001**	1.146 (0.548–2.309)	0.717

Pathological model	HR (95% CI)	*P*-value	HR (95% CI)	*P*-value	HR (95% CI)	*P*-value
*ISUP > 2*						
Univariable analysis	4.080 (2.764–6.023)	**< 0.0001**	3.742 (2.418–5.792)	**< 0.0001**	6.532 (2.474–17.248)	**< 0.0001**
Multivariable analysis	2.742 (1.811–4.152)	**< 0.0001**	2.654 (1.663–4.235)	**< 0.0001**	4.261 (1.541–11.770)	**0.005**
*Seminal vesicle invasion*						
Univariable analysis	5.253 (3.416–8.078)	**< 0.0001**	6.244 (3.629–10.743)	**< 0.0001**	4.016 (1.935–8.336)	**< 0.0001**
Multivariable analysis	2.368 (1.432–3.915)	**0.001**	3.038 (1.615–5.715)	**0.001**	1.565 (0.655–3.741)	0.314
*Positive surgical margins*						
Univariable analysis	2.277 (1.579–3.283)	**< 0.0001**	2.397 (1.559–3.686)	**< 0.0001**	1.989 (0.990–3.995)	0.050
Multivariable analysis	1.684 (1.130–2.510)	**0.010**	1.833 (1.148–2.924)	**0.011**	1.201 (0.545–2.645)	0.650
*Pelvic lymph node invasion*						
Univariable analysis	7.211 (4.348–11.960)	**<0.0001**	6.041 (3.269–11.163)	**< 0.0001**	10.500 (4.219–26.131)	**< 0.0001**
Multivariable analysis	2.912 (1.628–5.208)	**0.010**	2.073 (1.000–4.300)	0.050	5.626 (2.041–15.512)	**0.001**

*HR* hazard ratio, *CI* confidence interval; see also Table 1

Multivariable analysis in “Clinical model” has been adjusted for the variables included in Table 2

Multivariable analysis in “Pathological model” has been adjusted for the variables included in Table S3

## Discussion

In tertiary referral centers, clinically localized PCa in elderly patients is increasingly being treated with RARP with rates ranging between 7.5% and 26.8% [3, 5, 7–9, 14–16]. Although the procedure is safe, elderly patients are more likely to have unfavorable functional outcomes in terms of urinary incontinence and erectile disfunction rates compared with younger patients [4–6]. Elderly PCa patients are more likely to be diagnosed with high-risk disease and to have a lower overall survival [1, 2, 10]. In contemporary RARP series, older patients are also more likely to have unfavorable pathology that does not translate into an increased risk of biochemical recurrence [1–5, 9]. However, elderly patients may have a marginally significantly lower rate of cancer-specific survival [4]. Although elderly PCa patients are more likely to belong to the high-risk class and harbor unfavorable pathology, drawbacks to disease progression remain an unsettled issue in contemporary RARP series. Our study showed that elderly cases included almost one-third (27%) of the PCa population treated with RARP. Although elderly patients were more likely to have an impaired physical status (ASA score 3), this did not impact on LOHS and major postoperative complications. Moreover, although elderly patients were more likely to belong to the EAU high-risk class, older age was not an independent predictor of disease progression. However, elderly patients belonging to the high-risk class and/or having unfavorable pathology were more likely to progress compared with younger patients, suggesting an adverse impact of advanced age on these features. This is a novel finding in the literature dealing with such a subject. Moreover, these findings may explain the results reported by *Gurung and associates* who showed that higher pathological stage and pelvic LNI were detected in the older group who had lower cancer-specific survival than younger cases [4]. Also, our study agrees with the study of *Mandel and associates* who showed that in a non-contemporary cohort including open and robotic surgery, patients with advanced age > 75 years were more likely to have a biochemical recurrence and/or metastatic progression [17]. Our study confirmed the importance of assessing unfavorable pathology for predicting poor oncological outcome [18]. Our results indicated that the relationship between EAU high-risk and disease progression among elderly patients is different from that among younger patients. We cannot estimate the hazard ratio for EAU high-risk PCa without specifying the age group at which the comparison is being made. Thus, advanced age was an effective modifier for EAU high-risk disease for the risk of disease progression, which increased exponentially in the elder age group. Our study is the first one showing an effect modification of age on PCa progression and is representative of a contemporary RARP cohort reflecting real-world practice in a tertiary referral center. However, confirmatory studies are required.

Our results need explanations and interpretations. In our opinion, these findings might be explained by the fact that elderly patients are more likely to be exposed for longer intervals of time to the adverse effects of aggressive cancers that are more likely to progress for the cumulative sequences of genetic mutations. Also, elderly patients might have a compromised immune system that allows PCa to progress to systemic and uncontrolled disease. Although, the adverse impact of advanced age on disease progression might be sustained and explained by these patterns, controlled studies are required to test these hypotheses.

The results of our study have implications in actual clinical practice. Elderly patients elected to RARP are more likely to belong to the EAU high-risk category and to have unfavorable pathology that are independent predictors of disease progression. Although advanced age does not predict PCa progression, it adversely impacts on oncological outcomes when evaluated inside these unfavorable categories. Elderly patients belonging to the EAU high-risk should be counseled because of the increased risk of progression after surgery, which should be offered to selected and well-fit patients as a part of potential multimodal therapy in tertiary referral centers. Moreover, close follow-up is mandatory to detect and treat timely disease progression.

Our study has several limitations. First, it is retrospective and monocentric. Second, there was a preselection bias of elderly patients who were more likely to fit RARP surgery. Third, we applied the ASA score system, but not a geriatric assessment tool. Fourth, we did not perform a propensity-matched analysis to render more homogenous the study groups. Nevertheless, our investigation represents a contemporary RARP cohort reflecting real-world practice in a tertiary referral center.

## Conclusions

Elderly patients elected to RARP are more likely to belong to the EAU high-risk category and to have unfavorable pathology that are independent predictors of disease progression. Although advanced age does not predict PCa progression, it adversely impacts on oncological outcomes when evaluated inside these unfavorable categories. Elderly patients belonging to the EAU high-risk should be counseled about the increased risk of progression after surgery.

## Supplementary Information

Below is the link to the electronic supplementary material.

Supplementary file1 (DOCX 117 KB)
