# Supplementary material for: Advanced age portends poorer prognosis after radical prostatectomy: a single center experience
Source: Aging Clin Exp Res. 2022 Aug 17;34(11):2857–63. doi: 10.1007/s40520-022-02213-w (PMC9675672; doi:10.1007/s40520-022-02213-w)
Supplement: Supplementary file 1 — Supplementary file1 (DOCX 117 KB) [file 40520_2022_2213_MOESM1_ESM.docx]

**Tables**

| **Table S1. Pathological factors associated with senior age status in 901 prostate cancer patients treated with robot assisted radical prostatectomy** | | | | | | |
| --- | --- | --- | --- | --- | --- | --- |
|  | ***Age < 70 years*** | ***Age >/= 70 years*** | ***Univariate analysis*** | | ***Multivariate analysiis*** | |
| Number | 658 | 243 | OR (95% CI) | *P-value* | OR (95% CI) | *P-value* |
| Prostate weight; PW (grams; gr) | 50 (40,4 - 61) | 55 (44 - 70) | 1,017 (1,010 - 1,025) | **<0,0001** | 1,018 (1,010 - 1,026) | **<0,0001** |
| Tumor load; TL ( %) | 19,5 (10 - 30) | 19 (10 - 30) | 1,003 (0,994 - 1,012) | 0,559 | 0,995 (0,984 - 1,006) | 0,351 |
| ISUP grade group | | | | | | |
| ISUP < 3 | 368 (55,9) | 94 (38,7) | Ref |  | Ref |  |
| ISUP > 2 | 290 (44,1) | 149 (61,3) | 2,011 (1,489 - 2,717) | **<0,0001** | 1,961 (1,403 - 2,740) | **<0,0001** |
| Pathological stage (pT) | | | | | | |
| pT2 | 536 (81,5) | 170 (70) | Ref |  | Ref |  |
| pT3a | 58 (8,8) | 30 (12,3) | 1,631 (1,016 - 2,618) | **0,043** | 1,453 (0,875 - 2,412) | 0,149 |
| pT3b | 64 (9,7) | 43 (17,7) | 2,118 (1,387 - 3,235) | **0,001** | 2,011 (1,196 - 3,379) | **0,008** |
| Surgical margins; R | | | | | | |
| negative (R0) | 501 (76,1) | 184 (75,7) | Ref |  | Ref |  |
| positive (R1) | 157 (23,9) | 59 (24,3) | 1,023 (0,726 - 1,443) | 0,896 | 0,944 (0,646 - 1,379) | 0,766 |
| Lymph node invasion (pN status) | | | | | | |
| pN 0/x | 611 (92,9) | 219 (90,1) | Ref |  | Ref |  |
| pN1 | 47 (7,1) | 24 (9,9) | 1,425 (0,851 - 2,385) | 0,178 | 0,686 (0,373 - 1,264) | 0,227 |
| Legend: continuous variables are reported as medians (IQR, interquartile ranges) and categorical factors as frequency (percentage); ISUP, International Society of Urologic Pathology tumor grade group formulation; OR = odds ratio; CI, confidence interval. | | | | | | |

| **Table S2. Perioperative factors associated with senior age status in 901 prostate cancer patients treated with robot assisted radical prostatectomy** | | | | |
| --- | --- | --- | --- | --- |
|  | ***Age < 70 years*** | ***Age >/= 70 years*** | ***Univariate analysis*** | |
| Number | 658 | 243 | OR (95% CI) | *P-value* |
| Pelvic lymph node dissection (PLND) | | | | |
| no | 246 (37,4) | 83 (34,2) | Ref |  |
| yes | 412 (62,6) | 160 (65,8) | 1,151 (0,845 - 1,567) | 0,372 |
| High volume surgeon | | | | |
| no | 275 (41,8) | 116 (47,7) | Ref |  |
| yes | 383 (58,2) | 127 (52,3) | 0,786 (0,585 - 1,056) | 0,111 |
| Nerve sparing surgery | | | | |
| no | 436 (66,3) | 164 (67,5) | Ref |  |
| yes | 200 (30,4) | 65 (26,7) | 1,045 (0,721 - 1,514) | 0,815 |
| unknown | 22 (3,3) | 14 (5,8) | 1,186 (0,482 - 2,915) | 0,711 |
| Operative time; minutes | 220 (180 - 250) | 216 (180 - 250) | 1,000 (0,998 - 1,003) | 0,828 |
| Estimated blood lost; mL | 300 (200 - 450) | 250 (150 - 450) | 1,000 (0,999 - 1,000) | 0,506 |
| Clavien-Dindo complications | | | | |
| absent | 509 (77,4 ) | 171 (70,4) | Ref |  |
| grade 1 | 71 (10,8) | 37 (15,2) | 1,551 (1,006 - 2,393) | **0,047** |
| grade > 1 | 78 (11,9) | 35 (14,4) | 1,336 (0,865 - 2,063) | 0,192 |
| Length of hospital stay (days) | 4 (4 - 5) | 4 (4 - 6) | 1,046 (0,997 - 1,120) | 0,193 |
| Readmission after discharge | | | | |
| no | 638 (97) | 235 (96,7) | Ref |  |
| yes | 20 (3,0) | 8 (3,3) | 1,086 (0,472 - 2,499) | 0,846 |
| Legend: continuous variables are reported as medians (IQR, interquartile ranges) and categorical factors as frequency (percentage); ISUP, International Society of Urologic Pathology tumor grade group formulation; OR = odds ratio; CI: confidence interval. | | | | |

| **Table S3. Pathological factors prediciting prostate cancer (Pca) progression in 901 patients treated with robot assisted radical prostatectomy** | | | | | | |
| --- | --- | --- | --- | --- | --- | --- |
|  | ***No PCa progression*** | ***PCa progression*** | ***Univariate analysis*** | | ***Multivariate analysis*** | |
| Number of cases | 742 | 159 | OR (95% CI) | *P-value* | OR (95% CI) | *P-value* |
| Prostate weight; PW (grams; gr) | 50 (41,7 - 64,2) | 53 (42 - 64) | 1,004 (0,996 - 1,012) | 0,346 | 1,000 (0,992 - 1,009) | 0,939 |
| Tumor load; TL ( %) | 15 (10 - 30) | 20 (15 - 40) | 1,029 (1,015 - 1,031) | **<0,0001** | 1,002 (0,992 - 1,012) | 0,719 |
| ISUP < 3 | 423 (57) | 39 (24,5) | Ref |  | Ref |  |
| ISUP > 2 | 319 (43) | 120 (75,5) | 4,719 (3,278 - 6,794) | **<0,0001** | 3,480 (2,345 - 5,164) | **<0,0001** |
| pT2 | 618 (83,3) | 88 (55,3) | Ref |  | Ref |  |
| pT3a | 66 (8,9) | 22 (13,8) | 1,934 (1,211 - 3,088) | **0,006** | 1,000 (0,608 - 1,645) | 1,000 |
| pT3b | 58 (7,8) | 49 (30,8) |  |  |  |  |
| R0 | 586 (79) | 99 (62,3) | Ref |  | Ref |  |
| R1 | 156 (21) | 60 (37,7) | 2,279 (1,651 - 3,146) | **<0,0001** | 1,646 (1,148 - 2,361) | **0,007** |
| pN 0/x | 710 (95,7) | 120 (75,5) | Ref |  | Ref |  |
| pN1 | 32 (4,3) | 39 (24,5) | 5,549 (3,828 - 8,045) | **<0,0001** | 2,404 (1,559 - 3,707) | **<0,0001** |
| Legend: see Table 1; HR = hazard ratio; CI: confidence interval. | | | | | | |

**Figures**


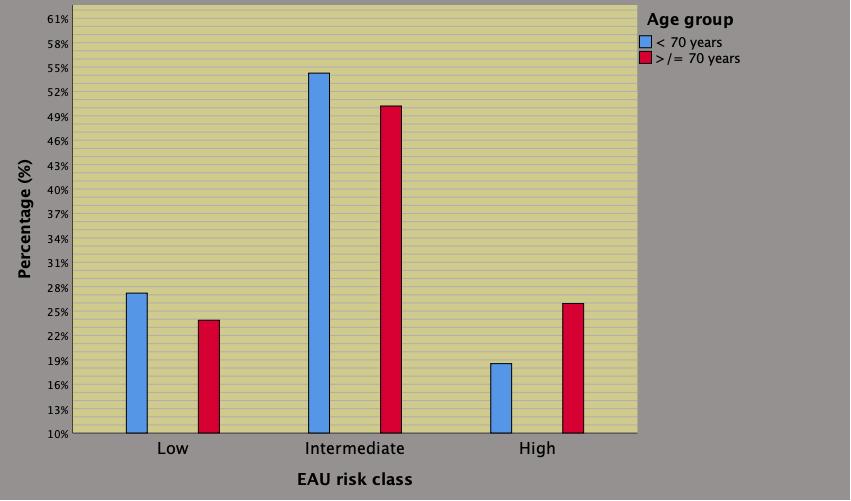


**Supplementary Figure 1**


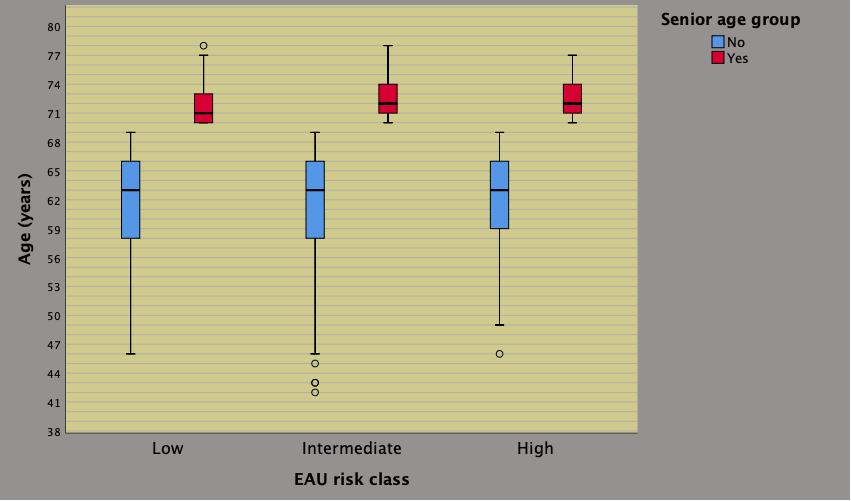


**Supplementary Figure 2**
